# Simple preparation of plant epidermal tissue for laser microdissection and downstream quantitative proteome and carbohydrate analysis

**DOI:** 10.3389/fpls.2015.00194

**Published:** 2015-03-27

**Authors:** Christian Falter, Dorothea Ellinger, Behrend von Hülsen, René Heim, Christian A. Voigt

**Affiliations:** Phytopathology and Biochemistry, Biocenter Klein Flottbek, University of HamburgHamburg, Germany

**Keywords:** laser microdissection, proteome analysis, cell wall, epidermis, plant–microbe interaction, infection structures, *Arabidopsis thaliana*

## Abstract

The outwardly directed cell wall and associated plasma membrane of epidermal cells represent the first layers of plant defense against intruding pathogens. Cell wall modifications and the formation of defense structures at sites of attempted pathogen penetration are decisive for plant defense. A precise isolation of these stress-induced structures would allow a specific analysis of regulatory mechanism and cell wall adaption. However, methods for large-scale epidermal tissue preparation from the model plant *Arabidopsis thaliana*, which would allow proteome and cell wall analysis of complete, laser-microdissected epidermal defense structures, have not been provided. We developed the adhesive tape – liquid cover glass technique (ACT) for simple leaf epidermis preparation from *A. thaliana*, which is also applicable on grass leaves. This method is compatible with subsequent staining techniques to visualize stress-related cell wall structures, which were precisely isolated from the epidermal tissue layer by laser microdissection (LM) coupled to laser pressure catapulting. We successfully demonstrated that these specific epidermal tissue samples could be used for quantitative downstream proteome and cell wall analysis. The development of the ACT for simple leaf epidermis preparation and the compatibility to LM and downstream quantitative analysis opens new possibilities in the precise examination of stress- and pathogen-related cell wall structures in epidermal cells. Because the developed tissue processing is also applicable on *A. thaliana*, well-established, model pathosystems that include the interaction with powdery mildews can be studied to determine principal regulatory mechanisms in plant–microbe interaction with their potential outreach into crop breeding.

## Introduction

The plant cell wall and its underlying plasma membrane are required to perceive environmental changes like biotic and mechanic stresses and also represent the first layers of defense to invading pathogens. In this regard, cells of the epidermal tissue have a predominant function as they form the outer barrier and are first in interacting with the surrounding environment. Epidermal plant cells usually differ in shape, function and physiological reactions to other plant tissues. Therefore, a specific analysis of separated epidermal tissue can provide new insights especially into the early regulation and organization of stress-related changes and adaption. This is of special interest in the field of plant–microbe interactions where cell wall thickenings, so-called papillae, are one of the most prominent and long-studied responses to invading pathogens in epidermal cells (de Bary, 1863). They contain the (1,3)-β-glucan callose as one of the most common chemical constituent, but also proteins (e.g., peroxidases and thionins), phenolics, and other putative antimicrobial and antifungal constituents (Aist and Williams, 1971; Sargent et al., 1973; Mercer et al., 1974; Sherwood and Vance, 1976; Mims et al., 2000). A precise, quantitative analysis of the composition of these stress-related structures and associated proteins would help to elucidate regulatory mechanisms involved in their organization and components required for establishing resistance to pathogens. However, conventional sample preparation from infected tissues, e.g., complete leaves after fungal infection, results in a relative strong dilution of the targeted stress-related defense structures because they only affect a relatively small part of the whole tissue. This is a major restriction in a time-resolved and plant line- or mutant-specific quantification of altered cell wall components, papillae constituents, and associated proteins.

To overcome these general limitations in sample preparation from tissues, laser microdissection (LM) techniques have been developed to separate specific cells or cellular structures from the surrounding tissue by using a focused laser (Emmert-Buck et al., 1996). After its initial application in medical research (Emmert-Buck et al., 1996; Fend and Raffeld, 2000; Wulfkuhle et al., 2001), LM has also been established in plant research to perform gene expression analyses from specific plant cell types (Asano et al., 2002; Nakazono et al., 2003; Casson et al., 2005), including epidermal cells (Inada and Wildermuth, 2005), and tissue selection for down-stream metabolic profiling (Schad et al., 2005b; Peukert et al., 2012), fluorometric assays (Jasik et al., 2011), or chemical analysis (Nakashima et al., 2008). Also in the area of plant–microbe interactions, an application of LM has been successfully demonstrated for the isolation of cells at infection sites and subsequent gene expression analyses (Chandran et al., 2010); and a growing number of reports about proteome analyses from LM-collected plant samples has been published (Fiorilli et al., 2012).

Despite the relatively large number of published LM studies including plant tissues, none of them provided a suitable protocol for our approach to separate stress-induced cell wall structures in epidermal cells for subsequent proteome and carbohydrate analysis in an efficient way. Methods described for LM of plant samples predominately include cross sections of either cryofixed of paraffin embedded tissue. Whereas paraffin embedding can be problematic for subsequent protein analysis (Ahram et al., 2003), cryofixation was shown to be compatible with subsequent protein analysis (Schad et al., 2005a), but is a relative elaborate method and difficult to apply on relative soft tissue, like leaf tissue from *Arabidopsis thaliana*. Therefore, we aimed to establish a LM method that would be relatively easy to operate and would not require tissue fixation. In addition, we wanted to avoid tissue cross section as early stage epidermal cell wall alterations, like papillae, would be difficult to identify, extending sample acquisition, and increasing possible protein degradation. A main prerequisite of our LM method development was to ensure an application on papillae generated in host epidermal leaf cells of the model pathosystem *A. thaliana* – powdery mildew, which would allow to study different mutants showing papillae formation and penetration resistance (Jacobs et al., 2003; Nishimura et al., 2003; Ellinger et al., 2013). Therefore, a primary target of our study was to develop an efficient method to isolate the plant’s adaxial epidermal cell layer containing localized, pathogen-induced cell wall structures. To reduce of sample contamination, we used LM coupled with laser pressure catapulting (LMPC) for sample collection (Meimberg et al., 2003) as a basic method in our study.

## Materials and Methods

### Biological Material

*Arabidopsis thaliana* (wild-type Columbia) as well as the powdery mildew *Golovinomyces cichoracearum* (strain UCSC1) used for leaf inoculation were cultivated and used as described by Stein et al. (2006). The cultivation of *Brachypodium distachyon* (inbred line Bd21) followed the description from Blümke et al. (2014). For all experiments, leaves from 4-weeks-old plants were used.

### Preparation of Single-Layered Epidermal Tissue by the Adhesive Tape – Liquid Cover Glass Technique

**FIGURE 1:**
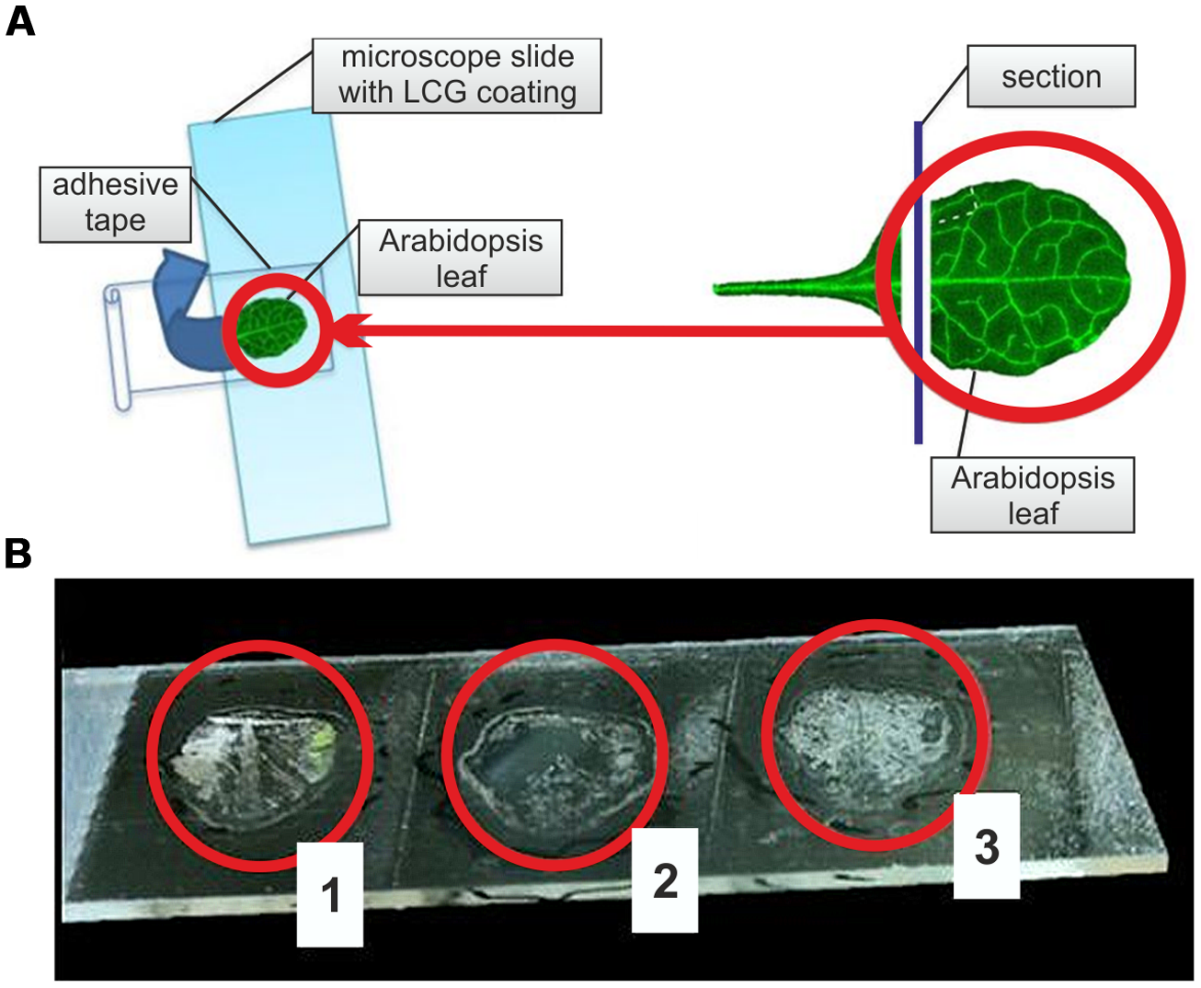
**Adhesive tape – liquid cover glass technique (ACT) for adaxial leaf epidermis preparation. (A)** Schematic overview of the application of ACT on *Arabidopsis thaliana* rosette leaves by sandwiching sectioned leaves between a microscope slide coated with liquid cover glass (LCG) and an adhesive tape. Blue arrow indicates direction of adhesive tape tear-off. Right panel demonstrates leaf sectioning before ACT. **(B)** Result of ACT preparation of adaxial *A. thaliana* leaf epidermis after three consecutive tear-offs of the same sample (1–3) on a LCG-coated microscope slide.

For the preparation of single layered epidermal leaf tissue, we developed a combined adhesive tape – liquid cover glass technique (ACT). First, microscope slides were coated with liquid cover glass (Carl Zeiss, Austria). To optimize adhesive surface capacity, slides were spray-coated with liquid cover glass, excess liquid removed, and after 2 min of drying again spray-coated. In case of a non-uniform drying of the liquid cover glass coat, which might impair epidermis preparation quality, a short heating of the coated slide at 30°C promoted the formation of an optimal adhesive surface. After a final drying period of 2–3 min, *A. thaliana* and *B. distachyon* leaf sections were placed with their adaxial side on the coated microscope slide. For the preparation of *A. thaliana* leaf sections, the petiole and about 1–2 mm of the adjacent part of the lamina were cut off; *B. distachyon* leaves were cut into 1 cm sections. Leaf sections on coated microscope slides were then laminated with a self-adhesive tape (Tesafilm transparent, product no. 57370, solvent-free adhesive on polypropylene foil, Tesa, Germany). During this procedure, the formation of air pockets should be avoided to obtain optimal results of epidermis preparation, which was achieved by a fast tear-off of the tape (**Figure 1A**). In case of an incomplete epidermis preparation, additional tear-offs of the adhesive tape containing the remaining leaf tissue on previously unused positions of the coated microscope slide allowed a preparation of the remaining epidermis (**Figure 1B**). ACT-prepared epidermal tissue layers were dried in a desiccator for 24 h to prepare samples for a long-term storage.

### Staining of Epidermal Tissue

To visualize pathogen-induced cell wall modifications, which contained the (1,3)-β-glucan callose, ACT-prepared epidermal tissue was stained with aniline blue [0.01% (w/v) in 150 mM K_2_HPO_4_, pH 9]. Small droplets of the aniline blue solution were directly placed on the tissue. During pipetting of the staining solution, the tissue should not float off because this would prevent a subsequent laser capture microdissection. Evaporation of water and drying of the tissue was done in a dark compartment for 16 h at room temperature (22–24°C) without humidity control (room humidity ranging from 40 to 60%). Aniline blue stained-cell wall structures of ACT-prepared epidermis were visualized via epifluorescence microscopy (Stein et al., 2006). Additional washing of the stained tissue samples was not required.

### Laser Capture Microdissection

For LM of ACT-prepared unstained or stained epidermal tissue, a PALM MicroBeam LM system (Carl Zeiss, Germany) was used, which was coupled with LMPC for sample collection. Typical settings for microdissection were a laser focus of 66% and a laser power of 32%, whereas the focus was re-adjusted to 64%, and the power to 62% during LMPC using the system’s operating software PALM Robosoftware V4.3 (Carl Zeiss, Germany). Optimal results were achieved by calibrating laser focus, laser power, and LMPC prior each single application. To establish LMPC from ACT-prepared epidermal tissue samples, disks with a diameter of 30 μm were randomly marked with the “close cut” function of the PALM Robosoftware. In case of aniline blue-stained samples, disks contained fluorescent regions that indicated pathogen-induced papillae. For optimal LMPC results, catapulting points were defined on a virtual circle with a diameter of 15 μm within the 30 μm disk using the “line auto-LPC” function of the PALM Robosoftware. Catapulted samples were collected in 40 μl denaturizing buffer [8 M urea, 2 M thiourea, 1 mM PMSF, 2% (v/v) protease inhibitor cocktail for plant cell and tissue extracts (Sigma–Aldrich, USA)], placed in the lid of collection tubes (Carl Zeiss). Collected samples were stored at -20°C until subsequent processing.

### Protein Purification of Dissected Epidermal Tissue Samples

Laser pressure catapulting-prepared epidermal tissue samples were sonicated in denaturizing buffer in the ultrasonic bath Elmasonic S40 (Elma Hans Schmidtbauer, Germany) for 1 min at 37 kHz, which was repeated three-times, including a short cooling of the sample on ice between each application. Proteins were separated from cell wall material and cell fragments by ultra-centrifugation at 4°C, 100,000 *g* for 60 min. The supernatant was used for protein analysis, the pellet for cell wall analysis. Before protein analysis by liquid chromatography coupled with tandem spectrometry (LC-MS/MS) using a nanoLC-electrospray iontrap MS/MS system [ion-trap, XCT, Agilent Technologies, Core Facility for Mass Spectrometric Proteomics, University Medical Center Hamburg-Eppendorf, Germany; mass spectrometric peptide identification followed the description in Hildebrand et al. (2013)], protein concentration of the sample solution was increased by methanol/chloroform precipitation (Wessel and Flügge, 1984), followed by conventional enzymatic digestion with trypsin. Mascot MS/MS Ion Search (Matrix Science, USA) was used for LC-MS/MS data analysis with a peptide mass tolerance of ±10 Da and a fragment mass tolerance of ±4 Da.

### Determination of Protein Concentration

After protein purification, protein concentrations of LMPC-prepared epidermal tissue samples were quantified using the fluorescence-based NanoOrange Protein Quantitation Kit (Life Technologies, USA) and the Synergy HT platereader (Biotek, USA) for detection. Procedures followed the manufacturer’s instructions.

### Mono-carbohydrate Composition by HPAEC-PAD

For cell wall analysis of ACT- and LCM-prepared epidermal tissue, the non-cellulosic mono-carbohydrate composition was determined via high-performance anion exchange chromatography with pulsed amperometric detection (HPAEC-PAD). The alcohol insoluble residue (AIR) was prepared from pellets deriving from previous ultra-centrifugation of sonicated epidermis samples. After destarching, samples were treated with trifluoro acetic acid (TFA) before HPAEC-PAD analysis. All procedures for AIR preparation, destarching, TFA treatment, and HPAEC-PAD application followed the description in Ellinger et al. (2013).

## Results and Discussion

### Leaf Epidermis Preparation by Adhesive Tape – Liquid Cover Glass Technique

**FIGURE 2:**
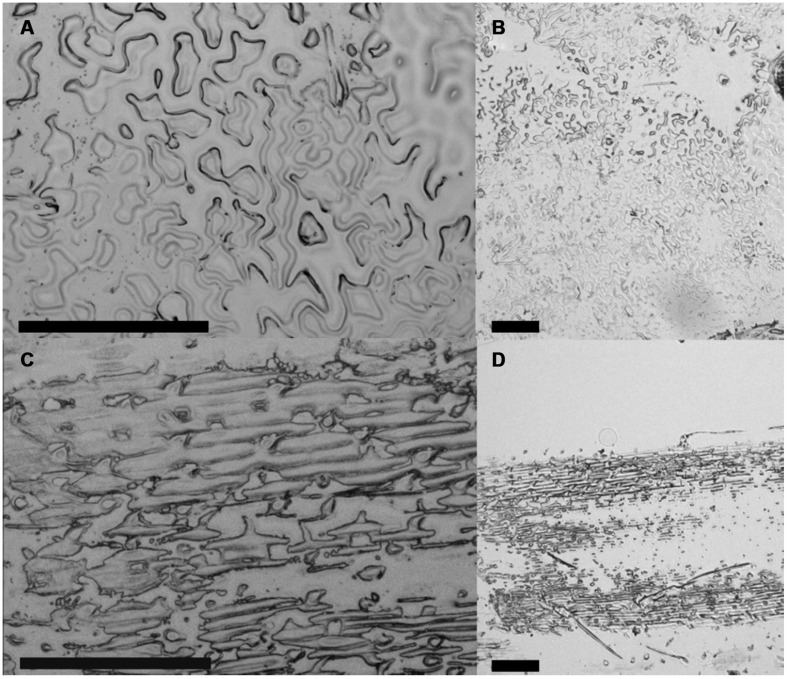
**Microscopic analysis of ACT-prepared adaxial leaf epidermis**. Epidermis preparation from 4-weeks-old **(A,B)** *A. thaliana* and **(C,D)** *B. distachyon* leaves using the ACT. Micrographs taken by the PALM MicroBeam laser microdissection (LM) system. Scale bars = 200 μm.

**FIGURE 3:**
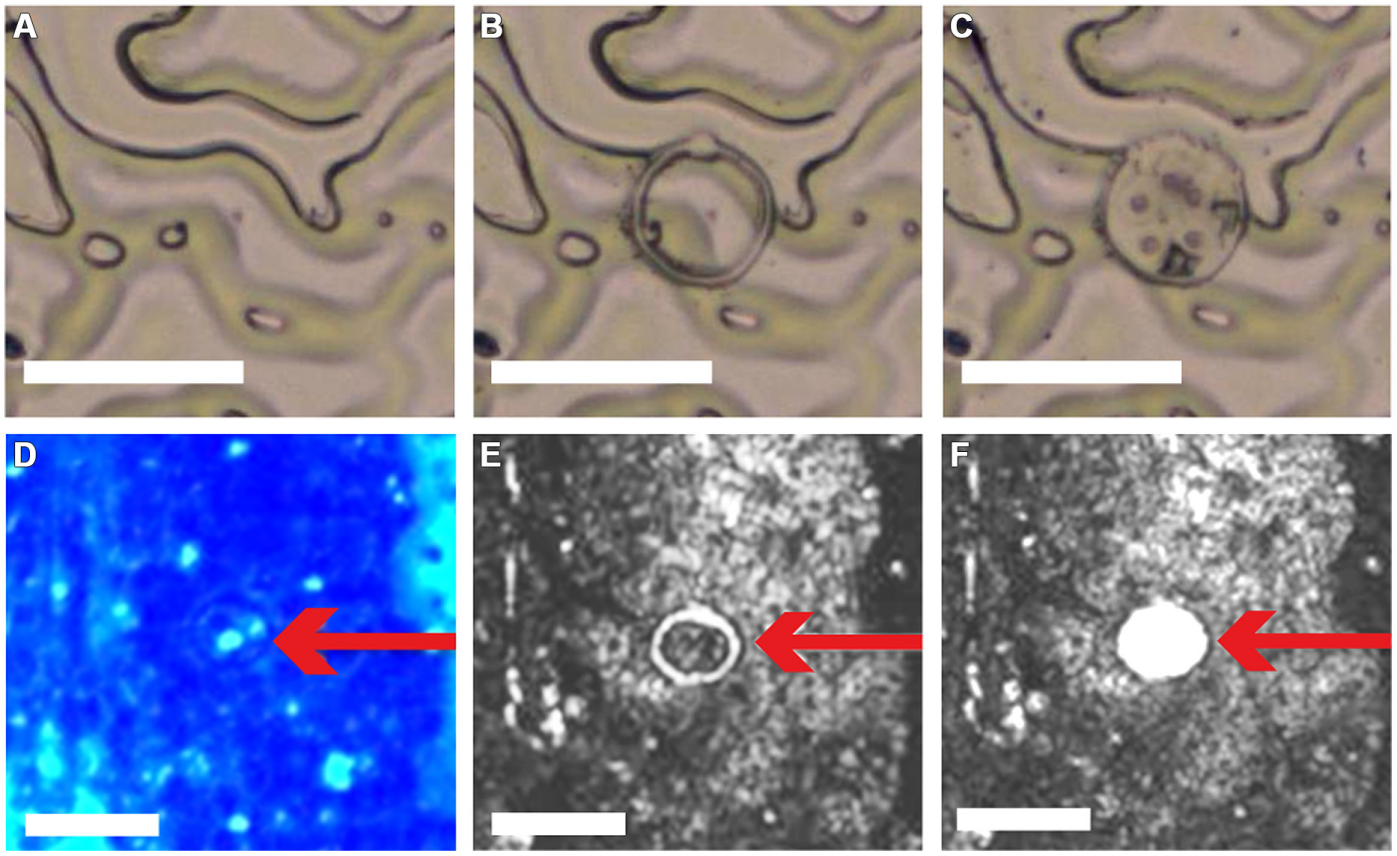
**Dissection and catapulting of 30 μm-disks from ACT-prepared adaxial leaf epidermis. Application of LM coupled with laser pressure catapulting (LMPC) on ACT-prepared *A. thaliana* adaxial leaf epidermis**. Epidermis **(A)** before and **(B)** after microdissection of a 30 μm disk; **(C)** removal of 30 μm-disk from epidermal tissue by LMPC. **(D)** ACT-prepared adaxial leaf epidermis 3 days post-inoculation with the powdery mildew *Golovinomyces cichoracearum*, additionally stained with aniline blue to visualize callose deposition. Red arrow indicates 30 μm-disk including pathogen-induced, callosic papillae (spots with light-blue fluorescence). **(E,F)** Bright-field micrographs of the same epidermis area as in **(D,E)** after microdissection and **(F)** after LMPC of a 30 μm-disk. PALM MicroBeam LM system used for LMPC and micrograph acquisition. Scale bars = 50 μm.

One of the main targets of our study was the establishment of a method for an easy preparation of the adaxial epidermis of plant leaves with a special focus on *A. thaliana*, characterized by relatively soft leaf tissue, which prevented an application of simple peeling techniques known from preparations of grass’ epidermal leaf tissue (Schultheiss et al., 2002). The developed ACT fulfilled our targets of an easy method of complete adaxial epidermis preparation without previous tissue fixation and applicability on soft tissues. The ACT method can be seen as further development of the perforated-tape epidermal detachment method, which was also applicable on *A. thaliana* adaxial epidermal tissue (Ibata et al., 2013), but not compatible with downstream LM applications. For epidermis preparation by ACT, we spray-coated microscope slides with liquid cover glass to generate an adhesive surface that is compatible with downstream LMPC applications (Moelans et al., 2011). After cutting off the petiole and lower part of the lamina, *A. thaliana* leaves were placed with their adaxial side on the coated microscope slide (**Figure 1A**). We used 1 cm leaf sections from the model grass *B. distachyon* as an additional reference for ACT epidermis preparation. Leaf sections were then laminated with an adhesive tape, sandwiching the plant sample between the coated microscope slide and the tape. A fast tear-off of the tape resulted in an epidermal cell layer, which was attached to the coated microscope slide. An additional tear-off of the adhesive tape containing the remaining leaf tissue on previously unused positions of the coated microscope slide allowed complete preparation of the epidermis (**Figure 1B**). However, three or more tear-offs of the same leaf sample increased the chance of a sample contamination with mesophyll tissue. Therefore, a microscopic examination of prepared epidermis sample was required to ensure sample collection only from regions with single epidermal cell layers. **Figure 2** provides an overview about successful single-layered, adaxial epidermis preparation from *A. thaliana* and *B. distachyon* leaf sections, showing the typical lobed profile of *A. thaliana* and the rectangular profile of *B. distachyon* epidermal cells. In addition, the result of epidermis preparation revealed that not the complete epidermal cell layer was attached to the coated microscope slide, but predominantly the outward-oriented part of the cells (**Figures 2A,C** and **3A**). This suggests that the cytosolic part of the epidermal cells was mainly removed during the tear-off of the adhesive tape. The observed effect of ACT promoted our efforts in analyzing the proteome of only stress-induced cell wall structures and associated plasma membrane fractions.

In the next step, we tested LMPC from ACT-prepared epidermal tissue choosing a disk size of 30 μm in diameter for dissection because this area would cover also enlarged papillae with extended callose deposition from powdery mildew-resistant *A. thaliana* lines (Ellinger et al., 2013). As expected from the relatively low thickness of the ACT-prepared epidermal samples (**Figure 2**), LMPC was not problematic (**Figures 3A–C**). Also additional staining of ACT-prepared epidermal samples with aniline blue, which is specific for (1,3)-β-glucan polymers (Evans et al., 1984), to visualize powdery mildew- induced, callose-containing papillae (**Figure 3D**) did not affect LMPC (**Figures 3E,F**). This proved the applicability of the developed ACT for isolating entire and stained stress-induced cell wall structures by LMPC from adaxial plant epidermal tissue.

Optimized laser settings for microdissection allowed a collection of up to 5000 disks per day if all targeted sample disks were first dissected and then catapulted and collected in the denaturizing sample buffer.

### Proteome Analysis of LMPC-Prepared Epidermis Samples

After successfully establishing ACT preparation of epidermal tissue with subsequent LMPC, we tested whether the collected samples would generally allow a proteome analysis. Moreover, we also wanted to evaluate the appropriate amount of dissected disks, which would be required to perform significant comparable proteome analysis of different samples. Therefore, we randomly dissected 11,000, 35,000, and 50,000 disks from ACT-prepared *A. thaliana* adaxial epidermal leaf tissue (**Figures 3A–C**), which was equivalent to a total area of 7.8, 24.7, and 25.4 mm^2^, respectively. Total protein extracts from these samples yielded 0.69 [ ± 0.19] μg protein per 10,000 disks and were analyzed by liquid chromatography coupled with tandem spectrometry (LC-MS/MS). Results from ACT- and LMPC-prepared epidermal leaf samples were compared with control protein samples from whole *A. thaliana* leaf tissue.

**FIGURE 4:**
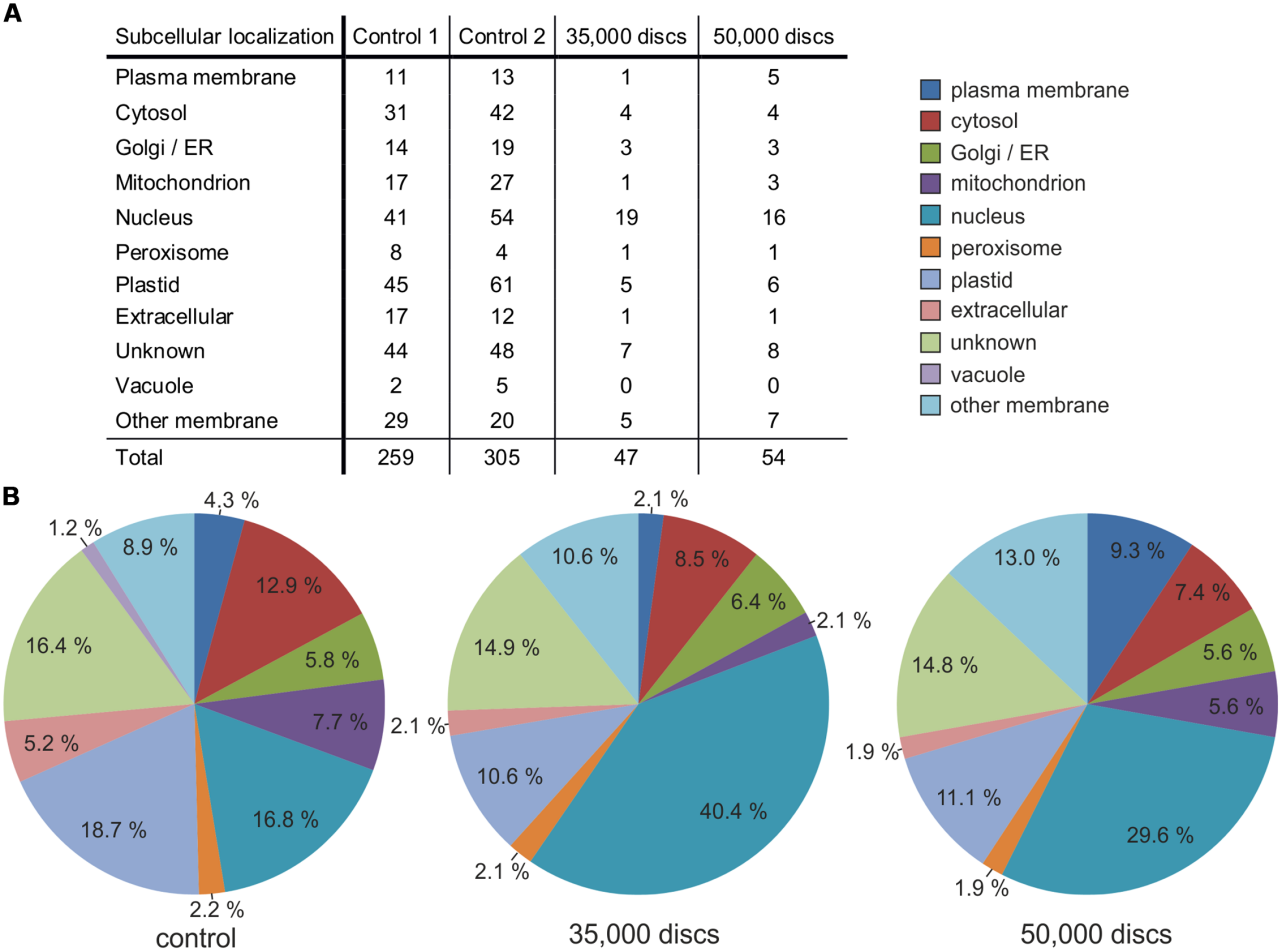
**Subcellular localization of extracted proteins from ACT-/LMPC-prepared adaxial *A. thaliana* leaf epidermis**. Epidermis preparation from 4-weeks-old *A. thaliana* leaves using the ACT with subsequent laser capture microdissection coupled with LMPC of 30 μm-disks; sample sizes: 35,000 and 50,000 disks. Protein extracts from whole leaf tissue served as control. Protein identity determined by LC-MS/MS. **(A)** Total number of different proteins and their subcellular localization in control and disk samples. **(B)** Relative share of each subcellular localization class based on data as shown in **(A)**. Control indicates the mean value of controls 1 and 2. A biological replicate gave similar results.

Whereas LC-MS/MS only identified 14 individual proteins in the sample deriving from 11,000 disks (0.38 μg protein analyzed), which we considered not sufficient for significant comparative analysis, 47 proteins derived from the 35,000-disks and 54 proteins from the 50,000-disks protein samples (1.21 and 1.73 μg protein, respectively; **Figure 4A**). Even though these numbers of proteins were only about a sixth of the amount detected in the control protein samples (5 μg protein; **Figure 4A**), they allowed a comparative analysis to evaluate the efficiency of the ACT epidermis preparation in accumulating proteins from the plasma membrane. A qualitative analysis of the subcellular localization of the detected proteins revealed that this was especially effective in the 50,000-disks sample. We observed a relative reduction of proteins originating from the cytosol, mitochondria, and plastids, whereas the relative fraction of proteins deriving from membranes, and especially the plasma membrane, was strongly enriched (**Figure 4B**). These results supported our microscopic observation that ACT of epidermal tissue results in a relatively efficient removal of the cytosol and cellular organelles. However, this method was not as effective in removing the nucleus from ACT-prepared epidermal samples as the relative fraction of nucleus-localized proteins was expanded in ACT-prepared epidermis samples (**Figure 4B**). Because the requested effects in accumulating plasma membrane-localized proteins and reducing proteins from the cytosol and organelles was not as distinct in the 35,000-disks sample as in the 50,000-disks sample, we propose sample sizes of about 50,000 disks (∼25 mm^2^) to be appropriate for downstream proteome analysis by LC-MS/MS. The observed effect of ACT epidermis preparation in accumulating membrane proteins was not only restricted to a qualitative analysis but also reflected in a quantitative analysis. A Top 15 list of protein abundance (**Table 1**) revealed a quantitative predominance of chloroplast localized proteins like ribulose bisphosphate carboxylase in the control sample from whole leaf protein extracts whereas the LMPC-prepared 50,000-disks epidermis sample was enriched in membrane and nuclear proteins. Remarkably, a (1,3)-β-glucosidase, which is known to be anchored to the plasma membrane (Elortza et al., 2003), was most abundant (**Table 1**).

**Table 1:** Protein abundance in control and ACT-/LMPC-prepared epidermal tissue samples (50,000 disks) from *Arabidopsis thaliana* leaves.

	Abundance	Locus	Description	Localization
**Control**	1	AtCg00480	ATP synthase subunit beta	Chloroplast thylakoid membrane
	2	At2g39730	Ribulose bisphosphate carboxylase/oxygenase activase	Chloroplast envelope
	3	AtCg00490	Ribulose bisphosphate carboxylase large chain	Chloroplast
	4	At5g26680	Flap endonuclease	Mitochondrion
	5	At3g12780	Phosphoglycerate kinase 1	Apoplast, cell wall, chloroplast, cytsosol
	6	At1g07950	Mediator of RNA polymerase II transcription subunit 22b	Nucleus
	7	At3g26650	Glyceraldehyde-3-phosphate dehydrogenase GAPA1	Chloroplast
	8	At4g29330	Derlin-1	Endoplasmic reticulum
	9	At4g02770	Photosystem I reaction center subunit II-1	Chloroplast thylakoid membrane
	10	At1g03130	Photosystem I reaction center subunit II-2	Chloroplast thylakoid membrane
	11	At4g28750	Photosystem I reaction center subunit IV A	Chloroplast thylakoid membrane
	12	At4g20360	Elongation factor Tu	Apoplast, chloroplast, nucleus
	13	At5g56140	KH domain-containing protein	Nucleus
	14	At4g04640	ATP synthase gamma chain 1	Chloroplast thylakoid membrane
	15	At1g35460	Transcription factor bHLH80	Nucleus

**50,000 disks**	1	At2g27500	Glucan endo-1,3-beta-glucosidase	Anchored component of plasma membrane
	2	At5g17690	Chromo domain-containing protein LHP1	Nuclear heterochromatin
	3	At3g04800	Translocase inner membrane subunit 23-3	Mitochondrial inner membrane
	4	AtCg00480	ATP synthase subunit beta	Chloroplast thylakoid membrane
	5	At1g56650	Transcription factor MYB75	Nucleus
	6	At3g49170	Pentatricopeptide repeat-containing protein	Chloroplast
	7	At2g40690	Glycerol-3-phosphate dehydrogenase	Chloroplast envelope
	8	At1g27050	Uncharacterized protein	-
	9	At5g62560	U-box domain-containing protein 41	Guard cell
	10	At3g06650	ATP-citrate synthase beta chain protein 1	Membrane
	11	At1g27660	Transcription factor bHLH110	Nucleus
	12	At5g45050	Probable WRKY transcription factor 16	Nucleus
	13	At3g02140	Ninja-family protein AFP4	Nucleus
	14	At5g35370	G-type lectin S-receptor-like serine/threonine-protein kinase	Plasma membrane
	15	At1g07650	Probable LRR receptor-like serine/threonine-protein kinase	Plasma membrane

These results from LC-MS/MS analysis ACT- and LMPC-prepared epidermal leaf samples from *A. thaliana* clearly showed that this method approach is appropriate for proteome analysis of selected cell wall structures and associated membranes. However, whereas the total number of proteins detected in control samples was in a range that can be expected in complex proteome analyses (∼280–300 different proteins in 5 μg total protein sample; Mallick and Kuster, 2010) with the system used in this study, protein numbers were below this expectation in ACT- and LMPC-prepared epidermal leaf samples. This suggests possible protein degradation during ACT-preparation and LM of epidermal leaf samples, when proteases were not specifically inhibited by protease inhibitors. To avoid possible protein degradation at this stage of sample preparation, addition of protease inhibitors to the liquid cover glass might provide an adhesive film with protease-inhibiting properties.

### Mono-carbohydrate Composition of LMPC-prepared Epidermis Samples

Besides proteome analysis, our target for developing a simple epidermis preparation method was to facilitate quantitative non-cellulosic mono-carbohydrate analysis of defined cell wall structures using high-performance anion exchange chromatography with pulsed amperometric detection (HPAEC-PAD). To test HPAEC-PAD on ACT-prepared epidermal leaf cell layers, we dissected 6,000 disks and extracted the mono-carbohydrates from the hydrolyzed hemicellulosic cell wall fraction. As a control, we applied the same method on LMPC samples from liquid cover glass-coated microscope slides without epidermis preparation.

**FIGURE 5:**
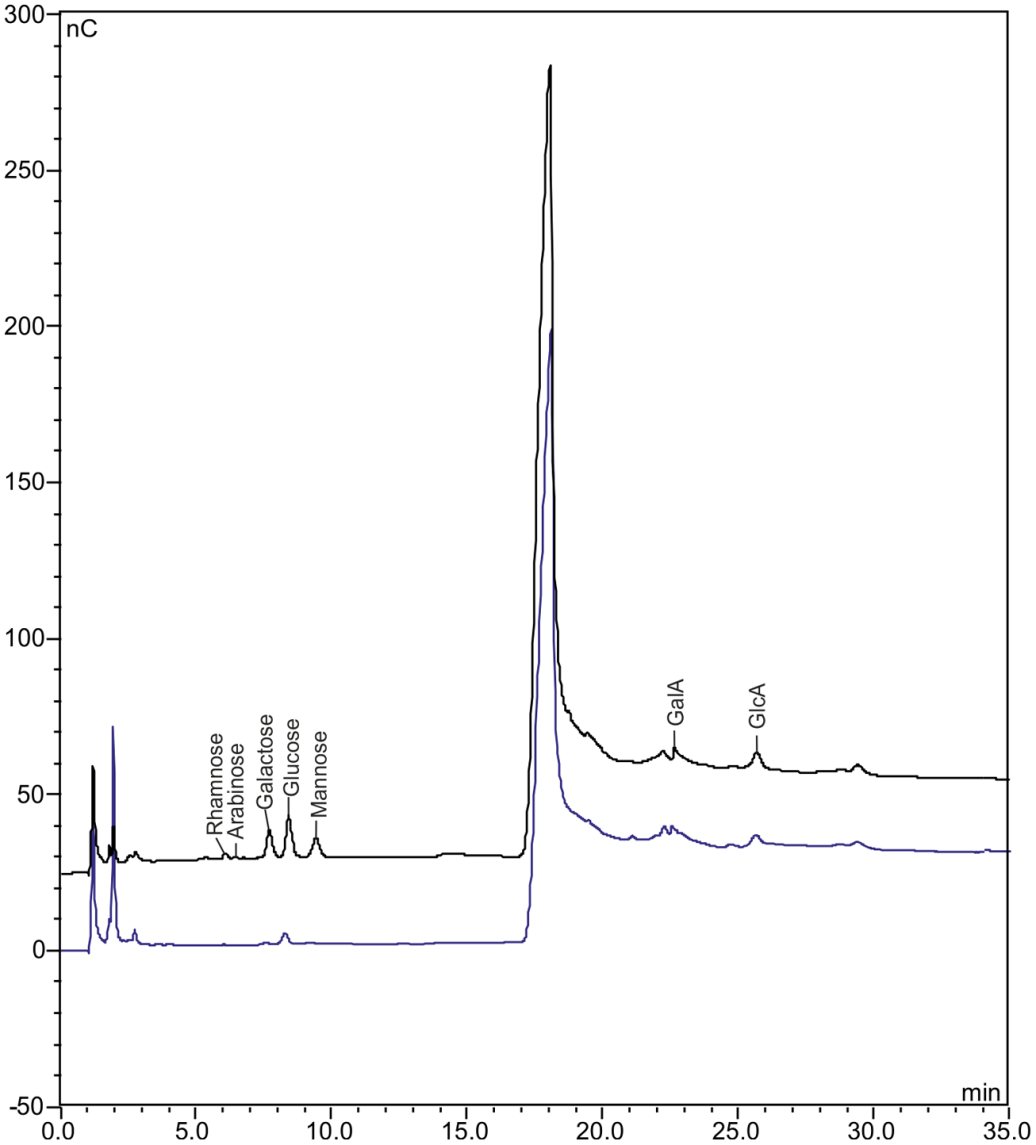
**Chromatograms obtained by HPAEC-PAD of ACT-/LMPC-prepared adaxial *A. thaliana* leaf epidermis**. Epidermis preparation from 4-weeks-old *A. thaliana* leaves using the ACT with subsequent laser capture microdissection coupled with LMPC of 30 μm-disks; sample size: 6,000 disks. Non-cellulosic mono-carbohydrate composition determined via high-performance anion exchange chromatography with pulsed amperometric detection (HPAEC-PAD). Black line: chromatogram of epidermal disk sample; blue line: chromatogram of control sample (6,000 liquid cover class disks, same treatment as epidermal disk sample). GalC, galacturonic acid; GlcA, glucuronic acid. A biological replicate gave similar results.

In the 6,000-disks sample, we detected the mono-carbohydrates galactose, glucose, mannose, galacturonic, and glucoronic acid that are typical for *A. thaliana* cell walls (Ellinger et al., 2013) in such amounts that would allow quantification. Only residual amounts were detectable for rhamnose and arabinose (**Figure 5**). We also detected residual amounts of glucose as well as galacturonic and glucoronic acid in the control liquid cover glass sample (**Figure 5**). Because it is most likely that these mono-carbohydrate contaminations derive from liquid cover glass, it would not be possible to avoid this contaminations based on the developed ACT for epidermis preparation. Hence, it would be required to analyze the same sample size of liquid cover glass-coating in parallel to plant samples to facilitate precise mono-carbohydrate quantification. In this regard, we consider a sample size of 6,000 disks (∼8 mm^2^) as a lower limit for significant and precise mono-carbohydrate quantification.

In addition to proteome analysis, the successful detection of hemicellulose-derived mono-carbohydrates clearly revealed that ACT- and LMPC-prepared epidermal leaf samples can be used for the quantitative analysis of the cell wall composition of defined cell wall structures using HPAEC-PAD.

## Conclusion

The developed ACT allows a simple preparation of complete adaxial epidermal tissue layers from large-scale leaf sections. Because the developed method is also applicable on soft tissue, preparations from the most important model plant *A. thaliana* have been facilitated. ACT epidermis preparation neither requires tissue embedding nor cross sections. Therefore, complete, defined cell wall structures or areas of modification can be readily identified and isolated using LMPC. This is of special importance in plant–microbe interactions where pathogen-induced cell wall modifications are decisive for plant defense. We successfully demonstrated further processing of ACT- and LMPC-prepared epidermis samples in downstream applications for proteome analysis and carbohydrate composition, opening new perspectives in the quantitative analysis of stress-induced cell wall modification.

## Author Contributions

CF, DE, BvH, RH, CV: conception and design of the research; CF, DE, BvH, RH: acquisition and analysis of the data; CF, DE, CV: interpretation of data; CF, DE, BvH, RH, CV: drafting the manuscript.

## Conflict of Interest Statement

The authors declare that the research was conducted in the absence of any commercial or financial relationships that could be construed as a potential conflict of interest.
